# Too Many Avoidable Suicides Occur Worldwide in Young Patients

**DOI:** 10.5041/RMMJ.10374

**Published:** 2019-10-29

**Authors:** Klaus Rose, David Neubauer, Jane M. Grant-Kels

**Affiliations:** 1 klausrose Consulting, Pediatric Drug Development & More, Riehen, Switzerland; 2 Department of Child, Adolescent and Developmental Neurology, University Children’s Hospital, Ljubljana, Slovenia; 3 Department of Dermatology, UConn Health, Farmington, CT, USA

**Keywords:** Suicidality, depression, pediatric drug development, children as “therapeutic orphans, ” pediatric investigation plan (PIP)

## Abstract

United States (US) and European Union (EU) laws attempt to counterbalance the presumed discrimination of children in drug treatment and drug development. The US Food and Drug Administration (FDA)-rewarded pediatric studies with antidepressants triggered in 2004 an FDA black-box warning of suicidality in young patients. Fewer antidepressants were prescribed, and the number of completed suicides of young persons increased. The dilemma between this warning and the need to adequately treat young depressed patients remains unsolved. We analyzed the history of drug development, the evolving view of diseases in young patients, US/EU pediatric laws, and pediatric studies triggered by FDA/European Medicines Agency (EMA) in depression and other diseases on the background of developmental pharmacology; financial, institutional, and other interests; and the literature. The FDA/EMA define children administratively, not physiologically, as <17 (FDA)/<18 years old (EMA). But young persons mature physiologically well before their 17th/18th birthday. Depression occurs in young persons, has special characteristics, but is not fundamentally different from adult depression. Young persons are not another species. Regulatory requirements for “pediatric” studies focus on “pediatric” labels. Many “pediatric” studies, including those in depression, lacked and lack medical sense and harm patients by placebo treatment although effective drugs exist. The FDA has partially abandoned separate “pediatric” efficacy studies, but not in psychiatry. Clinicians, parents, institutional review boards, and ethics committees should become aware of questionable “pediatric” studies, should re-evaluate ongoing ones, consider to suspend them, and to reject new ones. The concept of separate “pediatric” drug approval needs to be abandoned.

## INTRODUCTION

In 2003, a warning by the British Committee on Safety of Medicines cautioned against the use of paroxetine in children and adolescents under the age of 18 years to treat depression,^1^ after safety concerns had been reported by GlaxoSmithKline.^2^ Worldwide, regulatory authorities took up this warning in different ways.^3^ The United States (US) Food and Drug Administration (FDA) issued a black-box warning that antidepressants increase the risk of suicidality in young patients.^2^ The data that triggered these warnings came from 23 industry-sponsored pediatric antidepressant studies and the nationally funded US Treatment of Adolescents with Depression Study (TADS) that had investigated major depressive disorder (MDD), obsessive-compulsive disorder, and other psychiatric disorders. These studies resulted in a committee of clinicians, convened by the FDA, to discuss the findings.^2^ The black-box warning resulted in fewer prescriptions of antidepressants for young patients, less suicidality, but more completed suicides.^2^,^4^ This issue has been controversial from the beginning. The American Psychiatric Association (APA) expressed serious reservations that this warning might do more harm than good.^5^ The FDA-convened clinical committee’s recommendation for the black-box warning was not unanimous.^3^ Although regulatory authorities’ reaction worldwide was not uniform, they all essentially accepted the validity of the study data.^3^ Meanwhile, in the clinical world the position gradually emerged that treatment of young depressed patients is necessary and should be undertaken with appropriate caution.^3^,^6^–^8^

Some clinicians emphasized the usefulness of antidepressants in the treatment of pediatric depression.^9^ In addition to the APA, the American Academy of Child and Adolescent Psychiatry (AACAP) also criticized the black-box warning as not consistent with research and clinical experience.^3^ Today, there is general agreement that depressive patients of any age should not be left untreated, and that antidepressants are an important available therapy.^3^,^6^–^8^,^10^,^11^ Some clinicians: (1) recommend fluoxetine over other antidepressants,^12^ (2) suggest that further analyses of clinical trials data revealed an overall improvement of suicidality in young subjects treated with antidepressants,^13^ (3) claim that newer rating scales show similar rates of treatment-emergent suicidality in patients on antidepressants as placebo,^14^ and/or (4) do not mention the FDA black-box warning and the suicidality debate.^15^ Although some critical methodological comments were made,^3^ most publications have in common that they do not challenge the basic approach of the pediatric studies in antidepressants.^14^

In our view, discussing the used rating scales and other methodological details is not sufficient. The original 23 pediatric studies were FDA-rewarded with patent extensions, based on US pediatric legislation in 1997 that triggered pediatric studies in all clinical areas, including antidepressants.^16^ Today, the FDA is partially abandoning this concept in pediatric oncology, dermatology, and neurology, but not in psychiatry.^17^ Two pediatric melanoma studies were terminated; one was FDA-rewarded, and both were demanded by the European Medicine Agency (EMA). Both studies exposed young patients to sub-standard treatment inferior to approved adult treatment. Because physicians increasingly prescribed superior treatment off-label even to younger patients, recruitment had waned.^18^

The concept that children are “therapeutic orphans” was the intellectual basis of the US pediatric legislation.^19^,^20^ In our opinion, we need an analysis of this concept and of the resulting demand for “pediatric drug development.”^21^ This concept was also the basis of the pediatric studies undertaken with antidepressants. Do separate clinical studies in “children,” FDA-defined as persons <17 years old, make medical sense?^22^ Interestingly, simultaneously the FDA’s and scientists’ assessment of many childhood diseases has changed. For example, cancer in adolescents is no longer regarded automatically as “pediatric”; instead, the FDA recommends inclusion of adolescents into promising adult cancer studies.^23^ Additionally, epilepsy in children is no longer seen as a separate disease from adult epilepsy. For partial-onset seizures (POS), the FDA accepts “extrapolation” of efficacy from adults to young patients down to 4 years of age.^24^ The FDA also approved an ointment to treat atopic dermatitis based on studies in patients 2–79 years old without insisting on separate “pediatric” trials.^17^

## METHODS

Herein we trace the historical roots of the children-are-therapeutic-orphans concept, the imposure of the on-label/off-label framework on the newly created “pediatric population,” and its application on childhood depression and other diseases. We put this analysis into the framework of the history of drug development on the background of societal forces and institutional interests of US and EU pediatric laws that so far are barely addressed in the literature. On this background we explain why the FDA and the EMA insist on separate “pediatric” regulatory studies and discuss the consequences for treatment of depression in young patients.

## RESULTS

The concept of separate pediatric studies evolved gradually after the thalidomide disaster which triggered the US Drug Amendments Act of 1962;^25^ from 1962 on, the FDA approved drugs only on the basis of clinical studies performed before approval. The children-are-therapeutic-orphans concept and the demand for separate “pediatric drug development”^21^ are the result of the complex interplay of historically new institutions, including the FDA, with older centers of administrative influence, including the American Medical Association, the American Academy of Pediatrics (AAP), and others. The historical events that played a role include the following:

1. In the 1950s, toxicities had been observed in preterm newborns treated with antibiotics.^26^ Consequently companies inserted pediatric warnings into drug labels to prevent damage lawsuits in the litigious US.^17^,^18^,^20^ The first chairman of the AAP’s committee on drugs soon thereafter declared that these warnings denied children the use of many modern drugs and characterized children as “therapeutic orphans.”^19^
2. Before 1962, companies developed, produced, sold, and advertised new products with limited supervision. This now changed profoundly. New drugs were now required to have undergone clinical studies before FDA approval. Only FDA-approved drugs are allowed interstate commerce.^25^
3. In close collaboration between the AAP and FDA, the concept emerged that children need separate “pediatric drug development.”^21^,^26^ In 1979 the FDA defined “children” as <17 years old.^27^
4. The term “off-label” emerged in 1988, indicating the increased administrative power of the FDA.^28^ The on-label/off-label framework was soon imposed on the newly created “pediatric” population. As a result of this classification, a drug could be on-label in 17-year-olds and off-label in 16-year-old patients.
5. Since 1997, US law has rewarded industry-sponsored “pediatric” studies by 6 months patent extension. As of 2003, the FDA can also mandate “pediatric” studies without reward.^16^,^17^
6. “Pediatric drug development” became truly international when the European Union (EU) introduced its own pediatric legislation.^16^–^18^,^22^ Until 2020, the FDA cannot mandate “pediatric” studies in orphan diseases. The EMA has no such restrictions and has issued since 2007 over a thousand “pediatric investigation plans” (PIPs). A PIP is required for all new drugs, unless the targeted disease is listed as not existing in children (“class waiver” list).^16^,^18^,^22^

The children-are-therapeutic-orphans concept disregards that children’s bodies mature from birth on.^29^ It blurs two meanings of the word “child”: (1) all underage persons are legally “children”: they cannot give informed consent to participate in a study; and (2) physiologically, “children” are young, pre-pubertal, vulnerable persons. Adolescents and older pre-pubertal minors are physiologically no longer “children.” Justifications for separate “pediatric” studies switch between these meanings and confer an apparent physiological characteristic to the age limit of <17 (FDA)/<18 years (EMA).^17^,^18^,^20^,^22^ These age limits are administrative, not physiological.

To be sad is not a disease. Sadness becomes depression when it results in a loss of the patient’s relating to the external world. Depression can lead to suicide, a much more frequent cause of death in young patients than cancer.^30^ Major depressive disorder occurs in adults, adolescents, and older minors, but not in babies and infants. Until the 1960s it was thought not to occur in children of any age.^3^ Major depressive disorder is a clinically and etiologically heterogeneous disorder^31^ that requires a minimal maturity of the brain. Even authors that focus on the role of various transmitters and pathways involved in depression and new potential drug targets do not claim that below a certain age depression’s pathophysiology is fundamentally different.^32^ The concept of “pediatric drug development” did not evolve with scientific data in depression, but semantically as a blur between different meanings of the word “child” and furthermore from society’s desire to do “more” for “children,” as well as conflicts of interest that so far are little acknowledged.^18^,^22^ It originated from toxicities observed in preterm newborns treated with antibiotics.^17^,^18^,^20^,^22^,^27^ The FDA-rewarded “pediatric” trials in oncology, neurology, psychiatry, and other diseases did not emerge from clinical questions arising from hands-on clinical work, but from the concept that children-are-therapeutic-orphans.^17^,^18^,^20^,^22^

For pediatric researchers, “pediatric” trials offered sponsorship by pharmaceutical companies. Even if the outcomes were negative, the FDA rewarded these studies with 6 months patent extension.^33^ The study population was not defined scientifically, as the body does not change on the 17th or 18th birthday. The core challenge of the 23 FDA-rewarded “pediatric” depression studies and those organized thereafter is not based upon which rating scales were used, but the fact that there is no scientifically valid justification for separate efficacy studies of antidepressants in young patients defined by an administrative age limit. If depression was fundamentally different below a certain age, separate studies would be justified.

The FDA pediatric program contributed to the FDA’s standing in the world of medicine.^17^,^18^,^20^,^22^ Industry profited from the patent extensions.^33^

Table 1 lists currently recruiting “pediatric” studies with antidepressants. Table 2 shows that they all are triggered by regulatory demands.

**Table 1:** Pediatric Depression Studies Currently Recruiting.

No	NCT #	Abbreviated Study Title	Age (y)	# Pts/Centers
1	NCT03395353	Long-term S study of duloxetine hydrochloride in Japanese children and adolescents with depressive disorder	9–17	100/?
2	NCT03315793	DB S&E study of duloxetine hydrochloride versus placebo in Japanese children and adolescents with depressive disorder	9–17	148/?
3	NCT03665038	MC, DB, PC study on E, S, T, PK of brexanolone in adolescent female subjects with postpartum depression	15–17	80/13
4	NCT02709655	Interventional, R, DB, PC active reference (fluoxetine) fixed-dose study of vortioxetine in pts 7–11 y with MDD	7–11	600/133
5	NCT03569475	DB, PAC, S&E levomilnacipran ER study in pts 7–17 y with MDD	7–17	480/46
6	NCT02431806	DB, PAC, S&E levomilnacipran ER study in pts with MDD	12–17	660/91
7	NCT03185819	DB, R psychoactive PC, E&S study of 3 doses of IN esketamine plus comprehensive SoC for rapid symptom reduction of MDD, including suicidal ideation, in imminent risk for suicide	12–17	145/56

DB, double blind; E, efficacy; ER, extended release; IN, intranasal; MC, multicenter; MDD, major depressive disorder; NCT#, national clinical trial # in www.clinicaltrials.gov; PAC, placebo- and active controlled; PC, placebo-controlled; PK, pharmacokinetics; pts, patients; R, randomized; S, safety; S&E, safety and efficacy; SoC, standard of care; T, tolerability; y, year.

**Table 2:** Regulatory Origins of the “Pediatric” Studies in Antidepressants.

Compound	FDA	EMA
Duloxetine	FDA WR^34^	
Brexanolone	FDA WR^35^	
Vortioxetine	FDA PREA^36^	PIP EMEA-000455-PIP02-10-M04^37^
Levomilnacipran	FDA PREA^38^	
Esketamine		PIP EMEA-001428-PIP03-15^39^

PIP, pediatric investigation plan; PREA, pediatric research equity act: mandatory demand for pediatric studies; WR, written request: voluntary request that results in patent extension upon execution of the requested studies.

The inclusion criteria of the studies in Table 1 are administrative, not scientific. These studies were triggered by regulatory demands (see Table 2).

## DISCUSSION

The US pediatric legislation was introduced with high clinical expectations. The FDA mused in 2001: “Superior drug treatment information is expected to permit quicker recoveries from childhood illnesses, with fewer attendant hospital stays, physician visits and parental work days lost.”^40^ These were clear clinical endpoints. In depression, however, the FDA-rewarded separate “pediatric” studies and their intellectual processing achieved the contrary. It took decades for the recognition that depression exists in young persons. When the existence of depression in young persons was recognized, the children-are-therapeutic-orphans concept and the intellectual conceptualization of “pediatric” study data unfortunately resulted in the denial of effective antidepressants to young persons.

There is high merit in the FDA’s success in keeping dangerous compounds such as thalidomide from being sold as medicines. However, with the imposure of the on-label/off-label framework on the administratively created “pediatric” population, the FDA has laid the foundations for a development that increasingly withholds effective medicines from young patients. This was taken up and augmented by the EMA.^17^,^18^,^20^,^22^

The first US pediatric legislation of 1997 did not further advance pediatric oncology. It produced in single-agent-studies pharmacokinetics/pharmacodynamic data for many chemotherapy agents that had already been in clinical use for decades. Most studies were performed in heavily pretreated young patients that had already undergone several rounds of chemotherapy and were either refractory or relapsed. These studies provided regulatory coverage in hindsight for chemotherapy agents already known to be effective. Some got pediatric labels, others did not.^17^,^18^,^20^,^22^,^41^ They provided publications, networking, and funds for the involved researchers, patent protection for the sponsoring companies, and offered to the clinical pediatric research networks a new platform to discuss pediatric oncology that provided them easier access to pharmaceutical companies. A new decisive breakthrough in acute lymphoblastic leukemia (ALL) came not through pediatric legislation, but through successful involvement of the immune system in destroying malignant ALL cells by re-programmed leukocytes.^42^

Juvenile idiopathic arthritis (JIA) is not one disease, but an umbrella term for seven distinct diseases. They start in patients up to 16 years old, but they do not end at the 17th or 18th birthday. For the JIA clinical research networks, US and EU pediatric legislation provided many FDA-rewarded and then numerous EMA-demanded “pediatric” studies to study various JIA subtypes, subsequently documented in numerous academic publications.^43^,^44^ However, these studies were not “pediatric.” A disease that starts before the 16th year of age is not automatically “pediatric” or “juvenile,” as it can persist into adulthood. Juvenile idiopathic arthritis often continues into adulthood. Biologics and monoclonal antibodies (MABs) allow effective treatment, while steroids and methotrexate allowed symptom relief, but often with heavy side effects. Biologics, MABs, steroids, and methotrexate work before and after the 17th/18th birthday. Canakinumab, a human antibody against interleukin-1-beta (IL-1β), was first targeted against rheumatoid arthritis. It failed, but proved highly successful in cryopyrin-associated periodic syndrome (CAPS), systemic-onset JIA, and other disorders caused by overproduction of IL-1β.^45^ The pediatric rheumatology research networks Pediatric Rheumatology Collaborative Study Group (PRCSG) and Paediatric Rheumatology International Trials Organisation (PRINTO) praise US/EU pediatric legislation as responsible for today’s massively improved treatment of JIA.^43^ The reality is more complex. These groups started with commendable, legitimate studies in young patients when only limited treatment options were available. Several JIA sub-diseases are fundamentally different from adult rheumatoid arthritis. Many clinical JIA studies in young patients helped to improve treatment. However, this is not an issue of “pediatric” studies, but of the biology and the characteristics of the respective disease and the efficacy of newly developed medicines. The first pivotal CAPS studies were performed in patients of all ages, because CAPS is extremely rare. In other JIA sub-diseases, there are enough patients for large international trials.

When depression was recognized to exist also in young persons, treatment became jeopardized by the children-are-therapeutic-orphans concept and the imposure of the on-label/off-label framework in the administratively created “pediatric” population. The regulatory authorities demanded separate proof-of-efficacy studies in young patients despite the fact that, for example, there is no difference in post-partum depression between a 17- and an 18-year-old young mother. This represents an artificial separation (Table 1, study 3). The children-are-therapeutic-orphans concept led to the first 23 FDA-rewarded “pediatric” studies in antidepressants,^2^ followed by seven further studies,^14^ with the ongoing studies listed in Table 1, and contributed to psychiatrists’ and family doctors’ hesitancy to prescribe effective antidepressant treatment in young patients.

Depression in young patients is not fundamentally different from adult depression. Both represent an imbalance of feelings, based on an imbalance of receptors, transmitters, and connections in a brain that has reached some maturity and struggles to cope with reality. There are not two different types of depression, an adult one in patients ≥17/18 years, and a “pediatric” one in patients <17/18 years, nor two different types of antidepressants, those for patients <17/18 years old, and those for patients ≥17/18 years old. All depressed patients have the same receptors, transmitters, and imbalances. The suicidality discussion was triggered by the children-are-therapeutic-orphans concept, became a strong deterrent against effective treatment, and is probably responsible for many suicides that could have been avoided.

The controversy of alleged suicidality in young patients with MDD reflects complex societal challenges. The APA and AACAP concerns against the FDA black-box warning were appropriate,^3^ but they were expressed at a time when the FDA enjoyed a peak in administrative power and societal reputetion, supported by high enthusiasm about “pediatric drug development” in the medical world, the general public, the US congress, and in the pharmaceutical industry. This situation was further augmented by the EU pediatric legislation.^17^,^18^,^20^,^22^ Now, 15 years later, it is time to re-consider.

Apart from the “children-are-therapeutic-orphans” concept, the FDA developed and expressed additional concepts regarding different childhood diseases. Malignancies in “children” were declared to be fundamentally different from adult malignancies, with the exception of chronic myeloid leukemia.^41^ Chronic myeloid leukemia (CML) was the first malignancy for which a successful precision medicine, imatinib, had been developed; imatinib works in CML patients of any age. Separate “pediatric” studies in antidiabetic drugs were FDA-rewarded. Type 2 diabetes mellitus (T2DM) occurs in young persons, but is rare. One risk factor is obesity. All patients in the “pediatric” T2DM studies were massively overweight. All tested drugs reduced glycemia, but not all received a “pediatric” label.^17^,^18^,^20^,^22^ The FDA/EMA-triggered separate “pediatric” studies showed efficacy of insulin in type 1 diabetes mellitus.^46^ No chronological switch changes insulin receptors overnight at the 17th/18th birthday. Comparable “pediatric” studies were performed in dermatology, cardiovascular diseases, hypertension, and other diseases.^17^,^18^,^20^,^22^

Childhood depression, childhood cancer, and JIA are very different, but have in common that all new drugs need FDA/EMA approval; the submitted clinical studies must meet the authorities’ expectations. Underage patients in these three disease areas have very different needs. Treatment of cancer and JIA are straightforward once they are diagnosed, while depression is more complex. Psychiatrists and family doctors, insecure about the risks of antidepressants in young patients and in fear of being sued, hesitated to prescribe them, resulting in an increase of completed suicides.^2^,^4^ Why did the FDA not retract its black-box warning? Why could the APA not increase public pressure that would have forced the FDA to do so? And why is childhood cancer more discussed in the public than suicide?

Suicide, often resulting from depression, is a much more frequent cause of death than childhood cancer,^30^ but pediatric oncology attracts more public attention and funding. From 2020 on, the RACE for Children Act will give the FDA the authority to demand pediatric studies for new anticancer compounds.^47^ Today, the FDA recommends “basket” studies for pediatric cancer,^48^ but from 2020 on it will mandate studies—a mandate so far only given to the EMA.^17^,^18^,^20^,^22^

Clinical trials as the basis for drug approval have acquired an enormous weight in medical decision-making. Clinicians, however, are not guided only by the principles of “evidence-based medicine.” Clinicians do not execute regulatory authorities’ commands or recommendations, but are guided by codes of conduct that include their professional training, their being exposed to scientific, commercial, and other non-scientific influences, including real-life issues. Common sense is part of this background.

The exaggerated trust in clinical studies is ridiculed by Smith and Pell who demanded double-blind placebo-controlled studies to prove the efficacy of parachutes.^49^ Establishing a sound balance between clinical training, trials methodology, and common sense is demanding. It is time for the medical profession to re-establish a common-sense approach towards the regulatory authorities, in treating depression and other disorders.

The studies listed in Table 1 plan to recruit >2,000 young patients worldwide. All these studies, maybe with the exception of study 1, are unethical in our opinion and should be suspended by the responsible institutional review boards/ethics committees. In several clinical disciplines, critical voices have been published against regulatory-demanded “pediatric” placebo-controlled studies, including in multiple sclerosis^50^ and allergic rhinitis.^51^

Many FDA/EMA-demanded “pediatric” studies expose patients to substandard treatment and can potentially harm them. Furthermore, FDA/EMA positions provide an insufficient framework to treat depression and to prevent suicide in young patients. To improve treatment of depression and to prevent suicide will require us to overcome hidden conflicts of interest that have evolved in the past half century and are not yet recognized sufficiently.^17^,^18^,^20^,^22^

In dealing with depressed young patients, a common-sense approach and regulatory recommendations are divergent. Gradually, common sense has guided the clinical world to the conclusion that young patients also need effective antidepressant treatment, but the “suicidality” dilemma is not intellectually resolved. To resolve it, we propose to consider abandoning the children-are-therapeutic-orphans mantra.

The physicians that started to prescribe combination therapy for adolescents with conventional melanoma did so (and had to do so) off-label. Until these studies were terminated, precious time passed, and two “pediatric” melanoma studies recruited patients that would have deserved better treatment.^20^,^22^ We will never know how many young patients died of completed suicide because the regulatory authorities’ “suicidality” concept increased physicians’ hesitance to prescribe effective antidepressant treatment.

Institutional review boards/ethics committees should consider suspension of the studies listed in Table 1, and APA and AACAP should re-express their concerns against the FDA’s black-box warning of “suicidality” in young patients. Finally, US and EU pediatric legislation need revision.

## Abbreviations

AACAP: American Academy of Child and Adolescent Psychiatry
AAP: American Academy of Pediatrics
ALL: acute lymphoblastic leukemia
APA: American Psychiatric Association
CAPS: cryopyrin-associated periodic syndrome
CML: chronic myeloid leukemia
EMA: European Medicines Agency
EU: European Union
FDA: US Food and Drug Administration
IL-1β: interleukin-1-beta
JIA: juvenile idiopathic arthritis
MAB: monoclonal antibody
MDD: major depressive disorder
PIP: pediatric investigation plan
PRCSG: Pediatric Rheumatology Collaborative Study Group
PRINTO: Paediatric Rheumatology International Trials Organisation
T2DM: type 2 diabetes mellitus
TADS: Treatment of Adolescents with Depression Study
US: United States.
